# Effects of television viewing on brain structures and risk of dementia in the elderly: Longitudinal analyses

**DOI:** 10.3389/fnins.2023.984919

**Published:** 2023-03-08

**Authors:** Hikaru Takeuchi, Ryuta Kawashima

**Affiliations:** ^1^Division of Developmental Cognitive Neuroscience, Institute of Development, Aging and Cancer, Tohoku University, Sendai, Japan; ^2^Smart Aging Research Center, Tohoku University, Sendai, Japan; ^3^Department of Advanced Brain Science, Institute of Development, Aging and Cancer, Tohoku University, Sendai, Japan

**Keywords:** television, dementia, brain structures, NODDI, longitudinal

## Abstract

**Introduction:**

TV viewing in the elderly and in children is associated with subsequent greater decline of various cognitive functions including verbal working memory, but results of its association with subsequent risk of dementia were divided.

**Methods:**

In this longitudinal cohort study of UK Biobank, we investigated the associations of TV viewing length with subsequent risk of dementia and longitudinal changes of brain structural measures after corrections of a wide range of potential confounders.

**Results:**

Our results showed longer TV viewing was associated with increased risk of subsequent onset of dementia, as well as subsequent greater decline in intracellular volume fraction (ICVF) in the extensive areas of right lateral temporal cortex and the right medial temporal cortex, in the area around the left middle and inferior temporal cortex as well as the left fusiform gyrus, and the area adjacent to the left inferior frontal gyrus, and left insula.

**Discussion:**

These results may suggest prolonged TV viewing was associated with decline in density of neurites (axon, dendrites) in areas particularly implicated in language, communication, and memory, which are altered in dementia.

## Introduction

Television is an ubiquitous tool of modern life. However, its adverse effects on cognitive mechanisms have been well investigated. Longitudinal observational or intervention studies of children have shown that longer TV viewing is associated with subsequent declines in attention and cognitive ability, especially for verbal abilities including verbal working memory and verbal IQ (Gadberry, 1981; Sergeant et al., 2002; Zimmerman and Christakis, 2005; Landhuis et al., 2007; Takeuchi et al., 2015). In addition, recent large cohort studies of the elderly have linked longer TV viewing habits to subsequent declines in cognitive functions, including executive functions and visual and verbal short-term memory (Hoang et al., 2016; Bakrania et al., 2018; Fancourt and Steptoe, 2019). In the elderly, longer TV viewing has also been associated with an increased risk of cardiovascular disease (Grøntved and Hu, 2011). Moreover, our previous neuroimaging study involving children revealed that longer TV viewing is associated with greater increases in regional gray matter and lower developmental decreases in regional gray matter volume (rGMV) decreases in the fronto-polar areas (Takeuchi et al., 2015). In addition, ever since the association between TV viewing and dementia risk has been suggested (Aronson, 1993), a few studies investigated this association using small sample size and the results are divided and one reported the positive association and another failed to find the association (Lindstrom et al., 2005; Fajersztajn et al., 2021).

Despite these studies, the following issues have not been investigated; (a) associations between the length of TV viewing and subsequent changes in gray and white matter structural volume in the elderly, (b) the associations between the length of TV viewing and subsequent changes in brain microstructure properties of the brain, and (c) the associations between the length of TV and risk of dementia with the large sample size and corrections of a wide range of potential confounding variables. This study thus aims to investigate both of these issues. As longer TV viewing has been associated with greater declines in cognitive function of the elderly, we reasoned that this is likely to also increase the risk of dementia. Similarly, we predict that longer TV viewing is associated with lower brain volume, lower fractional anisotropy, and mean diffusivity, and other microstructural properties that are observed in aging and dementia in the fronto-polar areas, language and memory related areas. This is because TV viewing is consistently associated with declines of verbal functions and memory problems in the elderly (Takeuchi et al., 2015; Bakrania et al., 2018), and these neuroimaging characteristics characterize lower cognitive functions and dementia, and our previous study found effects of length of TV viewing in the fronto-polar areas in children (Nir et al., 2013; Takeuchi et al., 2015).

Identifying and describing the effects of TV viewing on cognitive decline, neural properties, and dementia development in the elderly is socially and scientifically important. For this study, we utilized data from the UK Biobank. Longitudinal design studies enable generation of associations between TV viewing and its subsequent impact on neurocognitive and neurological mechanisms in the aging brain.

To measure microstructural properties of the brain, we used metrics related to diffusion tensor imaging (DTI) and neurite orientation dispersion and density imaging (NODDI) in white matter (Edwards et al., 2017). Mean diffusivity (MD) describes the amount of water molecule diffusion regardless of direction, while axial diffusivity (AD) represents water molecule diffusion parallel to the tract within the voxel of interest and radial diffusivity (RD) measures the magnitude of water diffusion perpendicular to that tract. In addition, fractional anisotropy specifies the level of anisotropy of water diffusion. The intracellular volume fraction (ICVF) is used to represent neurite compartment density, as verified by histology in animal experiments (Sepehrband et al., 2015) while the isotropic volume fraction (ISOVF) reports the extracellular free water diffusion as well as the interstitial and cerebrospinal fluids (CSF). Finally, the orientation dispersion index quantifies the spread of fibers within an intracellular compartment. By combining these metrics with information on white matter volume, white matter changes can be comprehensively evaluated.

## Materials and methods

### Participants

**FIGURE 1:**
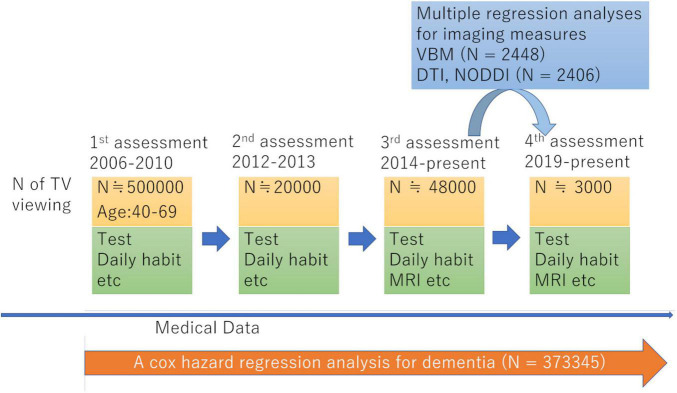
Study scheme.

For our study, we used data from the UK Biobank, which was obtained from a prospective cohort study of a middle-aged population in the United Kingdom and the procedures of which have been described elsewhere.^1^ Approval for these experiments was obtained from the North-West Multi-center Research Ethics Committee and written informed consent was obtained from each participant. Briefly, the participants went to one of 22 assessment centers throughout UK for data collection, with baseline data obtained from 502,505 participants. Our study included data for this cohort obtained at the first assessment visit (2006–2010), the first imaging data collection visit, which corresponded to the third assessment visit (2014–present), and the follow-up visit for imaging data collection, corresponding to the fourth assessment visit (2019–present). The schema of the study was presented in Figure 1. Sample of each analysis consists of those who have all the effective dependent and independent variables in the analyses.

### Assessment of time spent watching TV

Time spent watching TV was assessed by the following question: “In a typical day, how many hours do you spend watching TV?”. Answers were: “less than an hour a day” or any integer value between 0 and 24, with an answer of “less than an hour a day” was regarded as 0 h. In addition, Responses of more than 6 h were treated as 6 h in the analysis in line with those of recent representative studies of the effects of length of TV viewing (Hamer et al., 2017; Celis-Morales et al., 2018).

### Sociodemographic and lifestyle measurements used as covariates

Self-reported gender data was used. From the database, the neighborhood-level socioeconomic status at recruitment (cov1), education level at recruitment (cov2), household income (cov3), current employment status (cov4), metabolic equivalent of task hours (MET) (cov5), number in household (cov6), body mass index (BMI) (cov7), self-reported health status (cov8), and sleep duration (cov9) were extracted or calculated and included as covariates. For additional details, refer to the Supplementary methods section.

### Structural MRI acquisition and pre-processing for volumetric analyses

For the UK Biobank study cohort, MRI imaging data was obtained during the third and fourth assessment visits. Images were obtained from 3 imaging centers equipped with identical scanners (Siemens Skyra 3T running VD13A SP4 with a Siemens 32-channel RF receive head coil, Munich, Germany).

T1-weighted structural images were used for voxel-based morphometry analyses. Images were segmented and segmented gray matter and white matter were normalized using the diffeomorphic anatomical registration through exponentiated lie algebra (DARTEL) procedure, modulated, smoothed (8 mm full width at half maximum (FWHM)], and the resultant maps representing rGMV and regional white matter volume (rWMV) analyzed. Finally, the signal change in rGMV and rWMV between pre-(3rd visit) and post-(4th visit) images was computed at each voxel for each participant. In this computation, we included only voxels that showed GMV or rWMV values >0.10 in both pre- and post-images to avoid possible partial volume effects around the borders between different tissues. The resulting maps representing the rGMV and rWMV change between the pre- and post-MRI experiments (rGMV post–rGMV pre, rWMV post–rWMV pre) were then forwarded to the second level analysis, described in the next section. For details, see Supplementary methods.

For microstructure analysis, we used DTI and NODDI measurements released by the UK Biobank Imaging Study. Normalization was performed based on a previously validated protocol (Takeuchi et al., 2013). Briefly, diffusion images using the information of MD and FA, and modified DARTEL procedure which took account the FA signal distribution within white matter areas (to align images and tracts within white matter areas), were used to normalize all DTI and NODDI images as well as regional gray matter density (rGMD), regional white matte density (rWMD), and regional cerebrospinal fluid maps (rCSFD).

From the pre- and post-normalized images of the normalized MD, AD, RD, ICVF, ISOVF, OD maps, areas not strongly likely to be gray or white matter in our custom template (defined by “gray matter tissue probability + white matter tissue probability < 0.99”) were removed. Then, from the pre- and post-intervention normalized images of normalized FA map, areas not strongly likely to be white matter in our custom template (defined by “white matter tissue probability < 0.99”) were removed. Subsequently, normalized MD, AD, RD, ICVF, ISOVF, OD as well as FA images were smoothed by convolution with an isotropic Gaussian kernel of 8- and 6-mm full-width at half maximum, respectively. The images representing the baseline to the follow-up change of normalized smoothed images [e.g., ICVF (4th visit) − ICVF (3rd visit)] were used for the second level analyses described below.

For more details on these procedures, refer to the Supplementary methods. We did not use Tract Based Spatial Statistics (Smith et al., 2006), for many reasons, but the one of the reasons is that as far as we checked the values of diffusion measures in each tract in UK Biobank data that was generated by TBSS were generating apparent too many outliers, and we need to take the procedures we are used to correct these problems. The pre-processing method we used is shown to have validity, effectively solved all problems raised by voxel-based DTI measures raised by Smith et al. (2006), and generate analytical results that are similar to those generated by TBSS (Takeuchi et al., 2013).

### Psychological and non-whole brain imaging data analyses

Psychological and non-whole brain imaging data were analyzed using Predictive Analysis Software, version 22.0.0 (SPSS Inc., Chicago, IL, USA; 2010).

Cox proportional hazards models were used to examine the relationships between TV viewing length and dementia of all causes, as previously described (Lourida et al., 2019). All-cause dementia was ascertained using hospital inpatient records and linkage to death register data. This is a widely taken method in UK Biobank studies involving dementia (Lourida et al., 2019). For more details, see Supplementary methods. Participants with (a) self-reported dementia or Alzheimer’s disease or cognitive impairment without a diagnosis of all-cause dementia in either hospital inpatient records or death register data, (b) subjects already diagnosed with dementia at baseline or within 5 years after baseline, (c) those who died within 5 years after baseline, and (d) those with visuospatial memory performance lower than 2SD were excluded from the analyses The time scale considered spanned from the time of the first assessment visit and until 30 September 2021. Covariates were sex, age at the first assessment visit, values of cov1–cov9 at the first assessment visit, length of TV viewing at the first assessment visit, and visuospatial memory performance at the first assessment visit (for details, see Supplementary methods, fluid intelligence data was not available for a majority of subjects). For these analyses, we conducted both of analyses that treated the length of TV viewing as a continuous variable as well as analyses that treated the length of TV viewing as a categorical variable and separated 0–1 h, 2–3 h, 4–5 h, and 6 h or more, consistent with previous work (Celis-Morales et al., 2018). Although, we included a wide range of variables in this study, this study has a rich sample size, which mitigates the problem of overfitting (Riley et al., 2020).

### Imaging data analysis

Statistical analyses of imaging data were performed with SPM12.

Longitudinal whole-brain multiple regression analyses were employed to look for associations between TV viewing length at the third assessment visit and pre-(3rd assessment visit) to post-(4th assessment) scan changes of brain images [rGMV, rWMV, and DTI (FA, MD, AD, and RD) and NODDI images (ICVF, ISOVF, and OD)], with imaging data available from the third and fourth assessment visits. For rGMV and rWMV analyses, only voxels with a signal intensity of >0.05 for maps of subjects whose images were used to create the template were included for whole brain analyses. The analyses of DTI and NODDI maps were limited areas of the masks that were created above (white matter mask in the FA analysis and gray + white matter mask in other analyses).

In all imaging analyses, the independent variables were sex, age at the third assessment visit, the number of interval days between the third and fourth assessment visits, values of cov1–cov9 at the third assessment visit (except for cov1 and cov2, which refer to values at recruitment), head size ratio at the third assessment visit (calculated using UK Biobank output, in UK Biobank, normalization for head size is done by using T1-based “headsize scaling factor,” data id = 25,000, which is scaling factor estimated when transforming from native to standard space), and the length of TV viewing at the third assessment visit. We did not control the scanner site as was the case with the representative MRI studies of UK Biobank (Miller et al., 2016). There are more than 2 sites and it is difficult to model the effects in whole brain analyses. The time-lapse changes and site-specific effect may exist even when the scanners and parameters are identical, but there are no reasons to assume, those will confound the associations between TV viewing and imaging outcomes.

A multiple comparison correction was performed using threshold-free cluster enhancement (TFCE) (Smith and Nichols, 2009) with randomized (5,000 permutations) non-parametric testing using the TFCE toolbox.^2^ We applied a threshold of family-wise error corrected at *P* < 0.05.

## Results

### Basic data

Baseline psychological data for all participants is provided in Supplementary Table 1. TV viewing length at baseline was not associated with the covariates used in this study at the level greater than | *r*| > 0.4 at baseline. Among other covariates, there were no correlations among covariates that are greater than | *r*| < 0.4, except in the case of the association of age and current employment (*r* = −0.54). These results erase the concern of multicollinearity among psychological covariates. Note the associations between longer TV viewing length and longitudinal decline of non-verbal fluid reasoning and short-term numerical memory in UK Biobank have been previously reported (Bakrania et al., 2018).

### Prospective analysis of dementia

**FIGURE 2:**
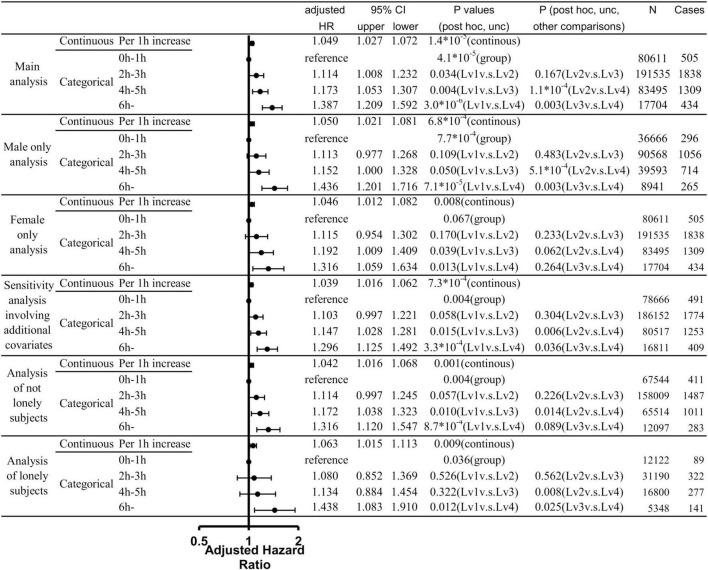
Statistical values and hazard ratios (HRs) with 95% confidence intervals (95% CIs) for the associations between length of TV viewing and all-cause dementia incidence from the main analyses and sub-analyses. Results of analyses that treated the TV viewing length as the continuous variable and analyses that treated the TV viewing length as the categorical variable were presented. *P*-values of each analysis, adjusted HR, cases and the participant of each category were presented.

Among the data of 502,505 participants in the present project, 121 participants had dementia record only through self-report and among remaining participants, 109 participants had dementia record that are diagnosed before the baseline date. Among the rest, 750 participants had dementia records diagnosed within 5 years after baseline, and 8,462 had died for other reasons during this period. Among the rest, after excluding who does not have data of any one of variables in the analysis, a total of 373,345 participants were included in our analysis. Among these patients, 4,086 cases of incident all-cause dementia were observed. Cox proportional hazard models in which TV viewing lengths were divided into four categories variables (0–1 h, 2–3 h, 4–5 h, 6 + h) revealed the overall group differences and compared with subjects with TV viewing lengths of 0–1 h at baseline, subjects with TV viewing length of 2–3 h, those of 4–5 h, those of 6 h or more. Further, compared with subjects with TV viewing lengths of 2–3 h at baseline, subjects with TV viewing length of 6 h or more showed higher risk. And compared with subjects with TV viewing length of 4–5 h at baseline, subjects with TV viewing length of 6 h or more showed higher risk. Statistical values and number of participants and cases in each group were presented in Figure 2.

Even if we use continuous variable for TV viewing length as are the cases of multiple regression analyses, the results showed the robust associations of TV viewing length with the subsequent risk of incident dementia [*p* = 1.4×10^–5^, increasing length of TV viewing by 1 h is associated with HR of 1.049 (CI: 1.027–1.072)]. These are not effects of group of suprathreshold and although we used 6 h as the longest TV viewing length based on the previous study and the fact that there are few subjects with longer TV viewing, using 8 or 10 h as the longest TV viewing also showed the association with subsequent risk of dementia [8 h: *p* = 3.8 × 10^–7^, increasing length of TV viewing by 1 h is associated with HR of 1.053 [CI: 1.032–1.074; 10: *p* = 1.3 × 10^–7^, increasing length of TV viewing by 1 h is associated with HR of 1.053 (CI: 1.033–1.074)].

We then conducted analyses limited to males and analyses limited to females to determine whether there is a difference in the relationship between TV viewing length and risk of dementia in males and females. When TV viewing length was treated as a continuous variable, there was a significant relationship between TV viewing length and risk of all cause dementia in both the male-only and female-only analyses; when TV viewing time was treated as a categorical variable, there were significant overall group differences only in the male-only analysis. However, a similar risk trend was observed in the analysis of women only. The statistical values were presented in Figure 2.

To resolve additional concerns that this association only reflects that prolonged TV viewing is a symptom of undiagnosed dementia or a reflection of serious medical and cardiovascular conditions, we further added analyses that included systolic blood pressure, tobacco smoking level, loneliness, baseline heart attack, angina, stroke, diabetes, cancer, other serious medical conditions, as covariates. This additional analysis also revealed the significant associations of TV viewing and subsequent onset of dementia and the effect size was comparable. The details were provided in Supplementary methods and Figure 2.

To further investigate the impact of loneliness, we stratified subjects who said they often felt lonely at baseline and those who did not and conducted analyses with same conditions as in the main analysis. The results showed similar effect sizes for both analyses, with the presence of significant overall group differences and significant continuous variable effects. The details were provided in Figure 2.

### Longitudinal brain imaging analysis

For brain imaging data analysis, using the data from the third and fourth assessment visits was used. A total of 2,448 and 2,406 participants were included in our analyses for volumetric analyses and microstructural property analyses (a part of entire UK Biobank projects’ participants participated in the MRI experiments of third and fourth assessments). The sample characteristics of the 2,448 subjects in volumetric analyses were provided in Table 1.

**TABLE 1:** Baseline characteristics of participants with and without incident dementia.

	No incident dementia (*n* = 369,259)	Incident dementia (*n* = 4086)
		**Mean**
Age	55.78(8.06)	63.97(4.83)
Townsend deprivation index	–1.44(2.99)	–1.14(3.19)
Education length	14.43(5.05)	12.91(5.23)
BMI	27.35(4.73)	27.81(4.86)
MET	31.76(35.53)	32.43(38.29)
Sleep length	7.15(1.03)	7.21(1.22)
Visuospatial memory (errors)	3.69(2.4)	4.39(2.51)
Systolic BP	137.17(18.43)	144.04(19.19)
		**Number**
**TV viewing length**		
(a) 0–1 h	80,106(21.7%)	505(12.4%)
(b) 2–3 h	189,697(51.4%)	1,838(45%)
(c) 4–5 h	82,186(22.3%)	1,309(32%)
(d) 6h-	17,270(4.7%)	434(10.6%)
Male number	173,437(47%)	2,331(57%)
**Household income**		
(a) Less than £18,000	75,786(20.5%)	1,748(42.8%)
(b) £18,000 to £30,999	92,650(25.1%)	1,280(31.3%)
(c) £31,000 to £5,1999	99,601(27%)	670(16.4%)
(d) £52,000 to £100,000	79,807(21.6%)	316(7.7%)
(e) Greater than £100,000	21,415(5.8%)	72(1.8%)
Currently employed	231,480(62.7%)	1,010(24.7%)
**Household number**		
(a) 1	68,506(18.6%)	1,068(26.1%)
(b) 2	166,959(45.2%)	2,432(59.5%)
(c) 3	59,026(16%)	375(9.2%)
(d) 4≤	74,768(20.2%)	211(5.2%)
**Overall health (4 levels)**		
(a) Poor	13,425(3.6%)	365(8.9%)
(b) Fair	72,264(19.6%)	1,158(28.3%)
(c) Good	217,800(59%)	2,127(52.1%)
(d) Excellent	65,770(17.8%)	436(10.7%)
**Current smoking level (3 levels)**		
(a) No	331,522(89.8%)	3,646(89.2%)
(b) Only occasionally	10,310(2.8%)	88(2.2%)
(c) On most or all days	27,321(7.4%)	350(8.6%)
**Often feel lonely**		
(a) No	299,972(81.2%)	3,192(78.1%)
(b) Yes	64,631(17.5%)	829(20.3%)
Diabetes*		
(X)	351,899(95.3%)	3,543(86.7%)
(O)	16,718(4.5%)	536(13.1%)
Heart attack*		
(X)	361,482(97.9%)	3,827(93.7%)
(O)	7,399(2%)	256(6.3%)
Angina*		
(X)	358,913(97.2%)	3,688(90.3%)
(O)	9,968(2.7%)	395(9.7%)
Stroke*		
(X)	364,330(98.7%)	3,881(95%)
(O)	4,551(1.2%)	202(4.9%)
Cancer*		
(X)	340,670(92.3%)	3,679(90%)
(O)	28,583(7.7%)	405(9.9%)
Other serious medical conditions*		
(X)	293,304(79.4%)	2,696(66%)
(O)	70,525(19.1%)	1,312(32.1%)

*Note these variables are not included in the main analysis and some of the participants included in the main analysis did not have these variables.

Whole brain multiple regression analyses using VBM analysis revealed there were no significant associations between TV viewing length at the third assessment visit and longitudinal changes in rGMV as well as rWMV from the third to the fourth assessment visit. There was a trend of positive association between TV viewing and longitudinal change in rGMV in the area of the ventromedial prefrontal cortex (*x* = −18, *y* = 36, *z* = −18, *t* = 4.24, cluster size = 1,269 mm^3^), which is close to the area where significant positive associations between TV viewing length at baseline and longitudinal change in rGMV was observed in our previous study of children (Takeuchi et al., 2015).

**FIGURE 3:**
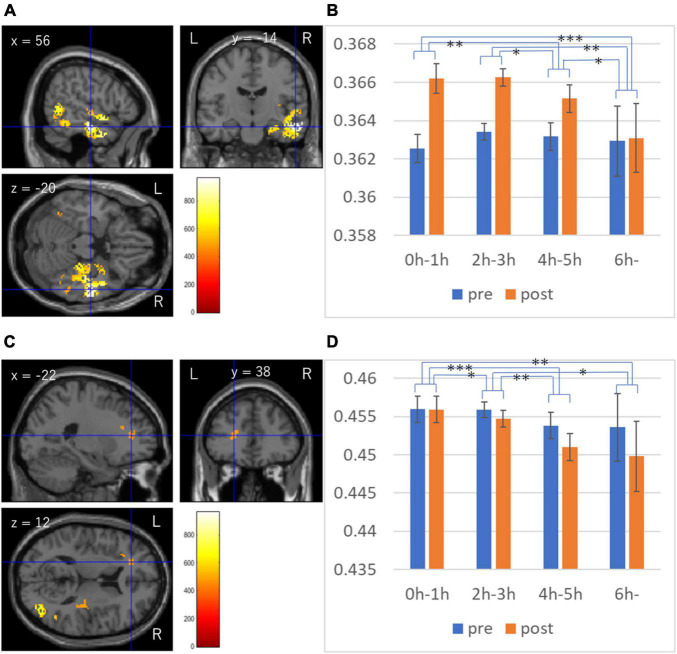
Associations between TV viewing length and longitudinal changes in intracellular volume fraction (ICVF). **(A,B)** Results are overlaid on a “single subject” T1 image from SPM. Results were obtained using a threshold of threshold-free cluster enhancement (TFCE) of *p* < 0.05 based on 5,000 permutations. The color represents the strength of the *t*-value. The color bar represents the TFCE score. It reflects both voxel’s height and the sum of the spatially contiguous voxels supporting it; therefore, it reflects both the strength and extent of effects. Profiles of imaging values at pre and post scans (which correspond to the third and the fourth assessment visits) in the significant cluster of the extensive cluster around the right temporal gyrus **(C)**, and the cluster in the white matter area of the left inferior frontal gyrus **(D)**. *p < 0.05, **p < 0.01, ***p < 0.001, in the *t*-tests comparing the raw pre to post differences of ICVF in the significant clusters between groups.

Whole brain multiple regression analyses involving DTI and NODDI analyses revealed significant results only in the analysis of ICVF. There were significant positive associations between TV viewing length at the third assessment visit and longitudinal changes in ICVF between from third to the fourth assessment visit in (a) the anatomical extensive cluster that spread in the areas involving the right amygdala, right hippocampus, right parahippocampal gyrus, right fusiform gyrus, right occipital lobe, right lateral temporal gyrus, right temporal pole, and right sagittal stratum and right stria terminalis, (b) an anatomical cluster that spread in the left inferior and middle temporal gyrus, and left fusiform gyrus, (c) an anatomical cluster that spread in the right Heschl gyrus, right insula, right putamen, right Rolandic operculum and right internal capsules, and (d) anatomical clusters that spread in the areas of left inferior frontal gyrus, left insula, and left anterior corona radiata (Figure 3).

Length of TV viewing was weakly but robustly negatively correlated with total brain volume that is normalized for head size at the third visit in this cohort (UK Biobank data field 25,009) after correction of age, sex and covariates 1–9 which were described in section “Materials and methods” (*p* = 1.8 × 10^–5^, *t* = 4.292, standardized beta = 0.020, *N* = 35949). Aside from the direction of causality of this cross-sectional association, we then investigate the impact of this effect (i.e., the impact of brain atrophy at the baseline (third visit) of brain imaging acquisition) on the above significant results of ICVF. We examined how inclusion of total brain volume that is normalized for head size at the third visit, changes the standardized beta of associations between TV viewing length and mean ICF values of significant clusters in the multiple regression analysis in addition to corrections of covariates of the main analysis. The results are presented in Table 2. In all significant clusters, the standardized beta were affected little by the inclusion of this covariate.

**TABLE 2:** Brain regions that exhibited significant negative correlations between length of TV viewing and changes in ICVF in longitudinal analyses.

Included gray matter areas* (number of significant voxels in left and right side of each anatomical area)	Included large bundles** (number of significant voxels in left and right side of each anatomical area)	x	y	z	TFCE value	Corrected *p*-value (FWE)	Cluster size (voxel)	Standardized beta (wiyhout TBV correction, with TBV correction)***
Amygdala (R:52)/Angular gyrus (R:4)/Cuneus (R:1)/Fusiform gyrus (R:331)/Heschl gyrus (R:1)/Hippocampus (R:128)/Inferior occipital lobe (R:74)/Middle occipital lobe (R:430)/Superior occipital lobe (R:32)/Parahippocampal gyrus (R:229)/Inferior temporal gyrus (R:774)/Middle temporal gyrus (R:642)/Temporal pole (R:89)/Superior temporal gyrus (R:223)/	Sagittal stratum (R:41)/Cingulum (R:19)/Heschl gyrus (R:19)/Stria terminalis (R:19)/Uncinate fasciculus (R:7)/	56	−14	−20	961.28	0.002	3490	−0.078, −0.077
Fusiform gyrus (L:12)/Inferior occipital lobe (L:2)/Inferior temporal gyrus (L:128)/Middle temporal gyrus (L:33)/	None	−50	−58	−8	532.14	0.032	177	−0.072, −0.073
Heschl gyrus (R:4)/Insula (R:34)/Putamen (R:6)/Rolandic operculum (R:4)/	Posterior limb of internal capsule (R:19)/Retrolenticular part of internal capsule (R:9)/External capsule (R:15)/	34	−20	14	492.6	0.041	104	−0.073, −0.073
None	Anterior corona radiata (L:16)/	−22	38	12	480.77	0.044	56	−0.077, −0.077
Inferior frontal triangular (L:4)/Insula (L:10)/	Anterior corona radiata (L:1)/	−30	28	8	468.25	0.047	15	−0.071, −0.071
None	Anterior corona radiata (L:10)/	−26	30	22	465.86	0.048	16	−0.071, −0.072

*Labelings of the anatomical regions of gray matter were based on the WFU PickAtlas Tool (https://www.nitrc.org/projects/wfu_pickatlas/) (Maldjian et al., 2003, 2004) and on the PickAtlas automated anatomical labeling atlas option (Tzourio-Mazoyer et al., 2002). Temporal pole areas included all subregions in the areas of this atlas.

**The anatomical labels and significant clusters of major white matter fibers were determined using the ICBM DTI-81 Atlas (http://www.bmap.ucla.edu/portfolio/atlases/ICBM_DTI-81_Atlas/).

***Standardized betas of the associations between TV viewing length and mean ICVF values of the significant clusters after corrections for covariates of the main analysis but without correction of total brain volume normalized for head size and those with correction of total brain volume normalized for head size.

## Discussion

Our study revealed new associations between the length of TV viewing and subsequent changes in ICVF of the brain, and dementia onset in the elderly. Consistent with our hypothesis, the longer TV viewing was associated with a slight but increased risk of dementia development. Brain imaging analyses revealed that longer TV viewing is associated with subsequent greater decline in ICVF in the extensive areas of right lateral temporal cortex and the right medial temporal cortex, in the area around the left middle and inferior temporal cortex, and the area adjacent to the left inferior frontal gyrus, and left insula. The significant decline of ICVF in these areas in ones with prior longer TV viewing were partly consistent with our hypothesis that longer TV viewing would be associated with alterations in the fronto-polar areas and areas relevant to the language and memory as described below and the direction of change is consistent with the aging-related changes in this measure (Cox et al., 2016). These changes were not attributed to physical activity levels or education levels, as this was controlled for in our regression analyses. These are not attributed to other diseases or symptoms of undiagnosed as dementia as well, as controlling for having cancer, diabetes, heart attack, angina, stroke, other serious medical conditions, or blood pressure and removed subjects who were diagnosed as dementia or died within 5 years after baseline does not impact the significance and effect size of findings of dementia. As subjects with particularly low memory functioning at baseline is removed in analyses of dementia these are also not attributed to effects of inaccurate reporting of subjects with high risk of subsequent incident dementia.

In the present study, among neuroimaging measures, the significant findings were only observed in analyses of ICVF. Decline in ICVF may reflect decline in density of neurites (axon, dendrites) (Deligianni et al., 2016). In addition, although, Direction of ICVF’s change may be not uniform, but ICVF generally declines in aging, and it is also reduced in patients with dementia, consistent with neural atrophy in aging and dementia (Nir et al., 2013). Decline in ICVF perhaps might be induced by the principle of use it or lose it in neural systems (Frick and Benoit, 2010; Shors et al., 2012), or the occlusion of capillaries due to lifestyle with prolonged TV watching. The reason why the significant reduction of diffusivity measures is not observed is clear, However, we speculate this could be due to a decrease of blood flow arising from a decline in brain activity or the subtle occlusion of capillaries, and reduction may be density of neurites may be cancelled. The latter possibility is supported by finding showing that even among the physically active elderly, reducing the length of TV viewing results in a reduced risk of cardiovascular disease (Patterson et al., 2020).

The associations between longer TV viewing and changes in ICVF were found in the areas relevant to language, communication, memory and so on. The largest cluster of the associations between longer TV viewing and decline in ICVF were found in the cluster that primarily spread in the right lateral temporal cortex and medial temporal cortex. Both of the right temporal and left temporal gyrus are suggested to play key roles in a number of verbal processes (Binder et al., 1994; Herholz et al., 1994; McGuire et al., 1996). However, the right one may more focus on non-verbal processes such as the processing of non-verbal sound discrimination, recognition and comprehension (McGlone and Young, 1992), processing of linguistic context (Kircher et al., 2001), irony and metaphor comprehension (Eviatar and Just, 2006), and gaze recognition (Akiyama et al., 2006). On the other hand, hippocampus and the parahippocampal gyrus are critically involved in memory processes (Squire and Zola-Morgan, 1991), and atrophy of these area is related to progression of Alzheimer’s dementia (Köhler et al., 1998). In addition, ICVF of the hippocampus is shown to be related to the accumulation of tau protein which is involved in the disease progression of Alzheimer’s dementia and memory function (Nir et al., 2020). The left fusiform has multiple functions, but the function relevant to here may be this region’s role in reading and letter recognition (Thesen et al., 2012). Finally, the significant association in the area in and adjacent to the left insula and left inferior frontal gyrus, may be related to the left insula’s function of speech processing, left inferior frontal gyrus’ function of process of word generation (Indefrey and Levelt, 2000; Oh et al., 2014), and important roles in the phonological loop of the working-memory system (Schulze et al., 2011). Through these neural changes of areas related to abovementioned functions, longer TV viewing may be related to the reduction of verbal abilities, memory functions and increased risk of dementia.

This study has a few limitations. First, this study is an observational longitudinal study and not an intervention study. Although we corrected for a wide range of potential confounding factors, including activity level (MET), socioeconomic status, baseline dependent variable values, sleep duration, BMI, household number, and health status in our analyses, still uncorrected pre-dispositions toward longer TV viewing may affect our outcomes. Alternatively, TV viewing length may be itself a sign of later neurocognitive changes. These issues could ultimately be solved through well-designed intervention studies. In addition, in this project, the contents of viewed TV programs, or how TV was watched was not assessed and effects of these may be a matter of future investigations.

Previously, longer TV viewing was associated with subsequent cognitive decline in the children and elderly, regional gray matter volume changes during child development, an increased risk of cardiovascular disease and divided results of altered risk of dementia. In this current study, we advanced understanding of the effects of TV viewing by showing that longer TV viewing in the middle to old age was associated with a decline in ICVF which is supposed to reflect density of neurites (axon, dendrites) in areas particularly implicated in language, memory and increased risk of subsequent risk of dementia after corrections of a wide range of confounding variables. Attention should thus be paid to elderly who have daily habits of watching TV for prolonged periods of time.

## Data availability statement

The datasets presented in this article are not readily available because the data is accessible upon the request to UK Biobank. Requests to access the datasets should be directed to https://www.ukbiobank.ac.uk/.

## Ethics statement

The studies involving human participants were reviewed and approved by North-West Multi-center Research Ethics Committee. The patients/participants provided their written informed consent to participate in this study.

## Author contributions

HT conceptualized the study, pre-processed, analyzed the data, and wrote the manuscript. RK played a key role in obtaining the relevant funding and supervised the study. Both authors contributed to the article and approved the submitted version.

## Footnotes

1
https://www.ukbiobank.ac.uk/media/gnkeyh2q/study-rationale.pdf

2
http://dbm.neuro.uni-jena.de/tfce/
