# Supplementary material for: Effects of television viewing on brain structures and risk of dementia in the elderly: Longitudinal analyses
Source: Front Neurosci. 2023 Mar 8;17:984919. doi: 10.3389/fnins.2023.984919 (PMC10030518; doi:10.3389/fnins.2023.984919)
Supplement: Supplementary file 1 [file Data_Sheet_1.docx]

**Supplementary online material**

**Supplemental Methods.**

**Additional information on subjects**

Adults of ages 40-69 years old who were registered with the National Health Service (NHS) and lived within 25 miles of the study's evaluation sites were invited by email to take part in the UK Biobank. No exclusion criteria were applied for this recruitment.

**Details on sociodemographic and lifestyle measures**

The following variables were calculated and used in this study. The descriptions of regarding these covariates were mostly reproduced from our previous studies using the similar covariates ([Takeuchi and Kawashima, 2021](#_ENREF_10)).

(cov1) Neighborhood-level socioeconomic status was measured by the Townsend index of material deprivation ([Townsend, 1987](#_ENREF_13)). Status was calculated based on the home postcode of the subject and represents a composite index of four postcode-level socioeconomic status variables: household overcrowding, unemployment, non-home ownership, and non-car ownership). A higher score implies a lower socioeconomic status. For this variable, only the value at recruitment was available and this value was used for all analyses.

(cov2) The education level was based on self-reported data. Education level categories of participant choices were transformed into numerical values based on previous study ([Okbay et al., 2016](#_ENREF_7)) as follows: “College or University degree” = 20 years; “A levels/AS levels or equivalent” = 13 years; “O levels/GCSEs or equivalent” = 10 years; “CSEs or equivalent” = 10 years; “NVQ or HND or HNC or equivalent“ = 19 years; “Other professional qualifications eg: nursing, teaching” = 15 years; “None of the above” = 7 years; “Prefer not to answer” = missing. For this variable, only the value at recruitment was available and this value was used for all analyses.

(cov3) The household income was the self-reported total income (before taxes) received by the subject's household. The available choices were <£18,000, £18,000 to £30,999, £100,000, £31,000 to £51,999, £52,000 to £100,000, >£100,000, do not know, and prefer not to answer. We converted these choices into ordinal variables of 1-5 (>£100,000 = 5) ([Shen et al., 2018](#_ENREF_9)) after excluding answers of do not know and prefer not to answer, as done elsewhere.

(cov4) The variable “current employment status” was used to describe the occupation information of the participants. The responses to the variable were: “In paid employment or self-employed”, “Retired”, “Looking after home and/or family”, “Unable to work because of sickness or disability”, “Unemployed”, “Doing unpaid or voluntary work”, “Full or part-time student”, and “None of the above”. Multiple responses were allowed. Subject responses were classified as either “In paid employment or self-employed” or not.

(cov5) Physical activity level was calculated from the recorded items from the International Physical Activity Questionnaire short form and was converted into a single measure of total physical activity in metabolic equivalent of task hours (MET). For more details, see the previous study ([Cullen et al., 2018](#_ENREF_3)).

(cov6) Participants were asked about the number of people in their household (including institutions such as care homes). Answers were assigned one of four variables: 1 (single person), 2 (two people), 3 (three people), and 4 (four or more people) as has been done elsewhere ([Sarkar et al., 2008](#_ENREF_8)).

(cov7) Body weight was measured using Tanita BC418MA scales. Height was measured using a Seca height measure. BMI was calculated from measured height and weight.

(cov8) Participants were asked about their health status, with possible answers being excellent, good, fair, poor that were converted to values of 4, 3, 2, and 1, respectively, before use in statistical analyses.

(cov9) Sleep duration was assessed with the item “About how many hours sleep do you get in every 24 h? (Please include naps.)” Responses were coded as integers and used in analyses after counting sleep durations of less than 3 hours into 3 and sleep duration longer than 10 hours into 10.

**Visuospatial memory task.**

Visuospatial memory was measured by the “pairs-matching’ task. In this test, participants were asked to memorize the positions of six card pairs, and then match them from memory while making as few errors as possible. Scores on the pairs-matching test are number of errors that participants made and therefore, higher scores reflect poorer cognitive functions.

**Ascertainment of dementia**

For the ascertainment of dementia of all causes, we followed methods established in a previous study ([Lourida et al., 2019](#_ENREF_5)), with descriptions in this subsection mostly being reproduced from this previous study and our previous study ([Takeuchi and Kawashima, 2021](#_ENREF_10)). All-cause dementia was determined based on hospital inpatient records containing data on admissions and diagnoses obtained from the Hospital Episode Statistics for England, Scottish Morbidity Record data for Scotland, and the Patient Episode Database for Wales. Additional cases were identified through death register data provided by the NHS Digital for England and Wales and the Information and Statistics Division for Scotland. Diagnoses were recorded using the International Classification of Diseases (ICD) coding system. Participants with dementia were identified as having a primary/secondary diagnosis (hospital records) or underlying/contributory cause of death (death register) using ICD-9 and ICD-10 codes for Alzheimer disease and other dementia classifications.

**Details of structural MRI acquisition and preprocessing for volumetric analyses**

For the UK Biobank study cohort, MRI imaging data was obtained for the third and fourth assessment visits. T1 weighted structural images were obtained from 3 imaging centers equipped with identical scanners (Siemens Skyra 3T running VD13A SP4 with a Siemens 32-channel RF receive head coil, Munich, Germany).

Structural images were acquired that contained straight sagittal orientations with a resolution of 1 × 1 × 1 mm and a field of view of 208 × 256 × 256, were taken over a duration of 5 minutes, and that contained 1-mm isotropic resolution generated using a 3-dimensional magnetization-prepared rapid-acquisition gradient echo.

For segmentation processes, outputs from the standard biobank processing pipeline involving FSL were used. Details of MRI protocols and segmentation are provided elsewhere ([Miller et al., 2016](#_ENREF_6);[Alfaro-Almagro et al., 2018](#_ENREF_1)).

Normalization processes were performed using Statistical Parametric Mapping software (SPM12; Wellcome Department of Cognitive Neurology, London, UK) implemented in Matlab (Mathworks Inc., Natick, MA, USA). First, using a segmentation algorithm implemented in SPM12, T1-weighted structural images of each scan were segmented, resulting in diffeomorphic anatomical registration through exponentiated lie algebra (DARTEL) import images. White matter segment images were generated through the standard biobank processing pipeline using FSL and were co-registered and resliced with DARTEL import images of white matter segments. Using the same parameters, gray matter segment images generated through the standard biobank processing pipeline using FSL were co-registered and resliced.

Using generated images, the DARTEL registration process was performed using SPM12 and both gray matter and white matter segmentation maps generated through the standard biobank processing pipeline using FSL were normalized to the Montreal Neurological Institute (MNI) space to give images with 3 × 3 × 3 mm^3^ voxels. The DARTEL template was then created using imaging data from the 250 baseline experiment images of subjects from the third assessment visit, as well as 250 follow-up images from the fourth assessment visit of different subjects. Next, using the existing template, DARTEL procedures were performed for all images. In addition, we performed a volume change corrections ([Ashburner and Friston, 2000](#_ENREF_2)). rGMV and rWMV images were then smoothed by convolving them with an isotropic Gaussian kernel of 8 mm full width at half maximum.

These processes were performed because although we used DARTEL procedures for precise registration processes, the segmentation quality of the standard biobank processing pipeline using FSL was better than that of SPM12’s new segmentation method and CAT 12.

**Details of diffusion MRI acquisition and preprocessing**

We used DTI and NODDI measurements released by the UK Biobank Imaging Study, including non-normalized FA, MD, AD, RD, ISOVF, ICVF, and OD maps. Details of the dataset can be found in the protocol documentation (<https://biobank.ctsu.ox.ac.uk/crystal/docs/brain_mri.pdf>) and in a previous study ([Miller et al., 2016](#_ENREF_6)). The key elements from these documents are described below.

Diffusion data were acquired using two b-values (b = 1,000 and 2,000 s/mm^2^) with a resolution of 2 × 2 × 2 mm and a field of view of 104 × 104 × 72 over a duration of 7 minutes, with a multiband acceleration factor of 3 in which three slices are acquired simultaneously. For each diffusion-weighted shell, 50 distinct diffusion-encoding directions were acquired covering 100 distinct directions over two b-values. Both diffusion tensor and NODDI models were fit voxel-wise and the image-derived phenotypes of various model outputs extracted from a set of white matter tracts. Tensor fits utilizing the b = 1000 s/mm^2^ data were used to produce maps including MD, FA, AD, and RD. The NODDI model was fit using the Accelerated Microstructure Imaging via Convex Optimization tool([Daducci et al., 2015](#_ENREF_4)) with outputs including ISOVF, ICVF, and OD.

Preprocessing and analysis of diffusion data were performed using SPM8 implemented in MATLAB. The following method descriptions have been largely reproduced from our previous study ([Takeuchi et al., 2013](#_ENREF_12);[Takeuchi et al., 2016](#_ENREF_11)). Using the b = 0 image, DTI and NODDI images were linearly aligned to the skull-stripped T2 image template of SPM8 to assist with the following procedures. When this registration processes did not proceed as expected, images were manually aligned to the template image.

A previously validated two-step segmentation algorithm for diffusion images and a previously validated DARTEL-based registration process were normalized ([Takeuchi et al., 2013](#_ENREF_12)) in a process involving FA signal distribution within white matter areas. All images, including segments of gray matter (regional gray matter density (rGMD) map], white matter (regional white matter density (rWMD) map], cerebrospinal fluid (CSF) (regional CSF density (rCSFD) map] of diffusion images, were normalized. The voxel size of these normalized images was 2 × 2 × 2 mm^3^. In these normalization processes, the template for the DARTEL process was created from 125 baseline (3^rd^ assessment visit) images and 125 follow-up (4^th^ assessment visit) images of different subjects.

The details of these procedures, which have also been described in our previous study ([Takeuchi et al., 2013](#_ENREF_12)), are as follows. Using the new segmentation algorithm implemented in SPM8, FA images of each individual subject were segmented into six tissues (first new segmentation). The default parameters and tissue probability maps were used for this process, except that affine regularization was performed using the International Consortium for Brain Mapping template for European brains and the sampling distance (approximate distance between sampled points when estimating the model parameters) was 2 mm. We then synthesized the FA image and MD map. In the synthesized image, the area with a WM tissue probability of >0.5 in the abovementioned new segmentation process was the FA image multiplied by −1 (hence, the synthesized image shows very clear contrast between WM and other tissues); the remaining area is the MD map (for details of this procedure, see below). The synthesized image from each individual was then segmented using the new segmentation algorithm implemented in SPM8 with the same parameters as above (second new segmentation). This two-step segmentation process was adopted because the FA image has a relatively clear contrast between GM and WM, as well as between WM and CSF, and the first new segmentation step can segment WM from other tissues. On the other hand, MD map has clear contrast between GM and CSF and the second new segmentation can segment GM. Since the MD map alone lacks clear contrast between WM and GM, we must use a synthesized image (and the two-step segmentation process).

We then performed a DARTEL registration process in SPM8. We used the DARTEL import image of the GM tissue probability map produced in the second new segmentation process as the GM input for the DARTEL process. The WM input for the DARTEL process was created as follows. First, the raw FA image was multiplied by the WM tissue probability map from the second new segmentation process within areas having WM probabilities of >0.5 (signals from other areas were set to 0). Next, the FA image * WM tissue probability map was co-registered and resliced based on the DARTEL import WM tissue probability image from the second segmentation, which created the DARTEL import image. The DARTEL template was created using imaging data from 125 baseline (3^rd^ assessment visit) experimental images of subjects and 125 follow-up (4^th^ assessment visit) experimental images of different subjects. Next, using the existing template, DARTEL procedures were performed for all images. The parameters for these procedures were changed as follows to improve accuracy. The number of Gauss–Newton iterations performed within each outer iteration was set to 10 and, in each outer iteration, we used 8-fold more timepoints to solve partial differential equations than the default values. The number of cycles used by the full multi-grid matrix solver was set to 8. The number of relaxation iterations performed in each multi-grid cycle was also set to 8. The resultant synthesized images were spatially normalized to MNI space. Using these parameters, the raw FA, raw MD, rGMD, rWMD, and rCSFD maps from the abovementioned second new segmentation process were normalized to give images with 1.5 × 1.5 × 1.5 mm^3^ voxels. The FA image * WM tissue probability map was used in DARTEL procedures because it includes different signal intensities within WM tissues and because the normalization procedure can take advantage of the intensity differences to adjust the image to the template from the perspective of the outer edge of the tissue and within the WM tissue. No modulation was performed in the normalization procedure.

We next created averages from the average images of normalized WM segmentation images (rWMD) of 250 images from which the DARTEL template was created, as described above, from the mask image consisting of voxels with a WM signal intensity >0.99. We then applied this mask image to the following normalized images of FA, therefore retaining from normalized images only areas that are highly likely to represent white matter. These images were then smoothed (6 mm full-width half-maximum) and then baseline to follow-up subtraction images were created and carried through to second-level analyses of FA.

We averaged normalized GM segmentation images (rGMD) and normalized WM segmentation images (rWMD) of 250 images from which the DARTEL template was created, as described above, from the mask image consisting of voxels with a GM signal intensity + WM signal intensity >0.99. We applied this mask image to the following normalized images (MD/AD/RD/ ICVF/ISOVF/OD), therefore retaining only areas that are highly likely to represent gray matter or white matter. These images were then smoothed (8 mm full-width half-maximum) and then baseline to follow-up subtraction images were created and carried through to second-level analyses of MD/AD/RD/ ICVF/ISOVF/OD.

**The rationales of choice of SPM versions**

We used different SPM versions for different preprocessing and analytic procedures. There are reasons for each of these choices.

First, SPM12 was used for preprocessing of T1 weighted structural images. This is simply because it is the newest and quality of corresponding preprocessing procedures confirmed by visual inspection was the best. Finally, SPM8 was used for preprocessing of DTI and NODDI images, because we used specifically developed preprocessing procedures and this was optimized and validate in SPM8 ([Takeuchi et al., 2013](#_ENREF_12)), and when the same approach was taken using SPM12, the quality of resulting images confirmed by visual inspection was inferior.

**Additional analyses for dementia that considered a wide range of additional variables**.

Although, analyses for dementia in the main text corrected the effects of physical activity level, we conducted additional analyses that removed participants who were diagnosed as dementia or died within 5 years after baseline and included a wide range of cardiovasucular and other serious diseases. This was to exclude the possibility that longer TV viewing reflects the undiagnosed dementia and longer TV viewing reflects lower cardiovasucular conditions or cardiovasucular diseases or other serious medical conditions. For these, the following variables were extracted from the database of UK Biobank and the values of these at baseline were added as covariates.

(cov10) Blood pressure was measured using a digital BP monitor (Omron), or a manual sphygmomanometer when the digital monitor was not available. Two readings or one reading were taken and we used the average, as has been done and described in a previous study ([Veldsman et al., 2020](#_ENREF_14)).

(cov11) Participants were asked about the current tobacco smoking status. Possible answers were 1 (No), 2 (Only occasionally), and 3 (Yes, on most or all days) and this was treated as a categorical variable. Responses of “prefer not to answer” were excluded.

(cov12) Participants were asked, "Do you often feel lonely?". Possible answers were 1 (No), and 2 (Yes) and this was treated as a categorical variable. Responses of “prefer not to answer” and “Do not know” were excluded.

(cov13-18) Participants were asked about the existence of doctor diagnosis of diabetes, heart attack, angina, stroke, cancer, and other serious medical conditions (item ID: 1049, 6150, 2453 2473). And the dichotomized variable of existence of each condition was generated based on this answer.

**References**

Alfaro-Almagro, F., Jenkinson, M., Bangerter, N.K., Andersson, J.L., Griffanti, L., Douaud, G., Sotiropoulos, S.N., Jbabdi, S., Hernandez-Fernandez, M., and Vallee, E. (2018). Image processing and Quality Control for the first 10,000 brain imaging datasets from UK Biobank. *Neuroimage* 166**,** 400-424.

Ashburner, J., and Friston, K.J. (2000). Voxel-based morphometry-the methods. *Neuroimage* 11**,** 805-821.

Cullen, B., Newby, D., Lee, D., Lyall, D.M., Nevado-Holgado, A.J., Evans, J.J., Pell, J.P., Lovestone, S., and Cavanagh, J. (2018). Cross-sectional and longitudinal analyses of outdoor air pollution exposure and cognitive function in UK Biobank. *Scientific reports* 8**,** 1-14.

Daducci, A., Canales-Rodríguez, E.J., Zhang, H., Dyrby, T.B., Alexander, D.C., and Thiran, J.-P. (2015). Accelerated microstructure imaging via convex optimization (AMICO) from diffusion MRI data. *NeuroImage* 105**,** 32-44.

Lourida, I., Hannon, E., Littlejohns, T.J., Langa, K.M., Hyppönen, E., Kuźma, E., and Llewellyn, D.J. (2019). Association of lifestyle and genetic risk with incidence of dementia. *Jama* 322**,** 430-437.

Miller, K.L., Alfaro-Almagro, F., Bangerter, N.K., Thomas, D.L., Yacoub, E., Xu, J., Bartsch, A.J., Jbabdi, S., Sotiropoulos, S.N., and Andersson, J.L. (2016). Multimodal population brain imaging in the UK Biobank prospective epidemiological study. *Nature neuroscience* 19**,** 1523-1536.

Okbay, A., Beauchamp, J.P., Fontana, M.A., Lee, J.J., Pers, T.H., Rietveld, C.A., Turley, P., Chen, G.B., Emilsson, V., Meddens, S.F., Oskarsson, S., Pickrell, J.K., Thom, K., Timshel, P., De Vlaming, R., Abdellaoui, A., Ahluwalia, T.S., Bacelis, J., Baumbach, C., Bjornsdottir, G., Brandsma, J.H., Pina Concas, M., Derringer, J., Furlotte, N.A., Galesloot, T.E., Girotto, G., Gupta, R., Hall, L.M., Harris, S.E., Hofer, E., Horikoshi, M., Huffman, J.E., Kaasik, K., Kalafati, I.P., Karlsson, R., Kong, A., Lahti, J., Van Der Lee, S.J., Deleeuw, C., Lind, P.A., Lindgren, K.O., Liu, T., Mangino, M., Marten, J., Mihailov, E., Miller, M.B., Van Der Most, P.J., Oldmeadow, C., Payton, A., Pervjakova, N., Peyrot, W.J., Qian, Y., Raitakari, O., Rueedi, R., Salvi, E., Schmidt, B., Schraut, K.E., Shi, J., Smith, A.V., Poot, R.A., St Pourcain, B., Teumer, A., Thorleifsson, G., Verweij, N., Vuckovic, D., Wellmann, J., Westra, H.J., Yang, J., Zhao, W., Zhu, Z., Alizadeh, B.Z., Amin, N., Bakshi, A., Baumeister, S.E., Biino, G., Bonnelykke, K., Boyle, P.A., Campbell, H., Cappuccio, F.P., Davies, G., De Neve, J.E., Deloukas, P., Demuth, I., Ding, J., Eibich, P., Eisele, L., Eklund, N., Evans, D.M., Faul, J.D., Feitosa, M.F., Forstner, A.J., Gandin, I., Gunnarsson, B., Halldorsson, B.V., Harris, T.B., Heath, A.C., Hocking, L.J., Holliday, E.G., Homuth, G., Horan, M.A., et al. (2016). Genome-wide association study identifies 74 loci associated with educational attainment. *Nature* 533**,** 539-542.

Sarkar, S.N., Huang, R.-Q., Logan, S.M., Yi, K.D., Dillon, G.H., and Simpkins, J.W. (2008). Estrogens directly potentiate neuronal L-type Ca2+ channels. *Proceedings of the National Academy of Sciences* 105**,** 15148-15153.

Shen, X., Cox, S.R., Adams, M.J., Howard, D.M., Lawrie, S.M., Ritchie, S.J., Bastin, M.E., Deary, I.J., Mcintosh, A.M., and Whalley, H.C. (2018). Resting-state connectivity and its association with cognitive performance, educational attainment, and household income in the UK Biobank. *Biological Psychiatry: Cognitive Neuroscience and Neuroimaging* 3**,** 878-886.

Takeuchi, H., and Kawashima, R. (2021). Diet and Dementia: A Prospective Study. *Nutrients* 13**,** Article 4500.

Takeuchi, H., Taki, Y., Hashizume, H., Asano, K., Asano, M., Sassa, Y., Yokota, S., Kotozaki, Y., Nouchi, R., and Kawashima, R. (2016). Impact of videogame play on the brain’s microstructural properties: Cross-sectional and longitudinal analyses. *Molecular Psychiatry* 21**,** 1781-1789.

Takeuchi, H., Taki, Y., Thyreau, B., Sassa, Y., Hashizume, H., Sekiguchi, A., Nagase, T., Nouchi, R., Fukushima, A., and Kawashima, R. (2013). White matter structures associated with empathizing and systemizing in young adults. *Neuroimage* 77**,** 222-236.

Townsend, P. (1987). Deprivation. *Journal of social policy* 16**,** 125-146.

Veldsman, M., Kindalova, P., Husain, M., Kosmidis, I., and Nichols, T.E. (2020). Spatial distribution and cognitive impact of cerebrovascular risk-related white matter hyperintensities. *NeuroImage: Clinical* 28**,** 102405.

**Supplemental Table 1.** Baseline characteristics of UK Biobank participants included in the present project (n = 502,505)

| Item | No. (%) | Mean (SD) | Range |
| --- | --- | --- | --- |
| Sex |  |  |  |
| Female | 273,382 (54.4) |  |  |
| Male | 229,122 (45.6) |  |  |
| Missing | 1 |  |  |
| Age, years |  | 56.5 (8.0) | 37-73 |
| Missing | 1 (0.0) |  |  |
| Length of TV viewing |  |  |  |
| 0, <1 h, 1h | 101,921 (20.3) |  |  |
| 2h, 3h | 249,659 (49.7) |  |  |
| 4h, 5h | 117,722 (23.4) |  |  |
| 6h or more | 27,788 (5.5) |  |  |
| Missing | 5,416 (1.1) |  |  |
| Average total household income before tax |  |  |  |
| Less than £18,000 | 97,198 (19.3) |  |  |
| £18,000 to £30,999 | 108,177 (21.5) |  |  |
| £31,000 to £5,1999 | 110,772 (22.0) |  |  |
| £52,000 to £100,000 | 86,266 (17.2) |  |  |
| Greater than £100,000 | 22,929 (4.6) |  |  |
| Missing | 77,164 (15.4) |  |  |
| Townsend index of material deprivation |  | -1.3 (3.1) | -6.25-11.00 |
| Missing | 624(0.1) |  |  |
| Employment status |  |  |  |
| In paid employment or self-employed | 287,149(57.1) |  |  |
| Not in paid employment or self-employed | 212,404(42.3) |  |  |
| Missing | 2,952(0.6) |  |  |
| Highest education qualification (years) |  | 13.95(5.1) | 7-20 |
| Missing | 10,113(2.0) |  |  |
| Fluid intelligence |  | 6.0(2.2) | 0-13 |
| Missing | 339,748(67.2) |  |  |
